# The Waste of Water

**Published:** 1898-09-17

**Authors:** 


### THE WASTE OF WATER.

No improvement has taken place in the water supply in the East of London, although, as the officials of the water company maintain, a very large amount of water is being daily pumped into the district. The question that occurs to everyone is—What becomes of all this flood of water? and the *Times* answers without hesitation, "It is wasted." The inference is suggested that the company has done its duty when it has pumped into the district an amount of water which, judging by the experience of many other places, ought to be amply sufficient for all purposes. We cannot, however, accept so simple a view of the matter. The duty of a company is to supply the individual householder, not the district at large, and the statements which are current as to the considerable quantity of water now being supplied only go to prove that the arrangements made by the company for its distribution and for the prevention of waste are utterly defective. These are matters over which the consumers have no control, and it is idle to throw on that vague body, "the public," the blame for the waste of water. However careful a householder may be, the sight of a neighbour going unpunished, though his taps are always running, is an education in wastefulness for which the company is directly responsible.

What is probably true is that, during the nine months in the year during which water is plentiful, it is cheaper to pump more water than it is to compel more carefulness. Thus habits of wastefulness are developed and fixed among the people, and it is futile to imagine that on the occurrence of drought all these habits, ingrained by custom in the daily lives of hundreds of thousands of people, can suddenly be turned into habits of thrift and carefulness by sending round the bellman, and distributing a few handbills.

The constant control of waste is no doubt expensive, and requires a considerable staff of inspectors, but the statements which have lately appeared in the papers as to the continued waste of water in certain parts of the East London district, statements made with the object of shifting the responsibility on to the public, are sufficient of themselves to show that the arrangements made for the detection and control of waste are absolutely inoperative. A year's education in carefulness ought to make a recurrence of the present condition of affairs extremely unlikely, if not impossible, even if not another gallon of additional supply were obtained.

In any case, however, such an education in carefulness will shortly be absolutely necessary if widespread water famines are not to become annual incidents in London life. The basis on which the Royal Commission on the Water Supply of London grounded its Report was that a supply of 35 gallons per head was enough. But many of the companies are even now supplying far more than that quantity, and huge populations are being brought up in the habit of using every drop of it, and it requires no prophet to see that if the present waste of water is allowed to go on we shall find ourselves face to face with water bankruptcy far sooner than the Royal Commission thought would be the case. Yet even this most optimistic Commission refused to cast its prophetic eye further ahead than the year 1931.

